# Development and Validation of a Diabetic Retinopathy Screening Modality Using a Hand-Held Nonmydriatic Digital Retinal Camera by Physician Graders at a Tertiary-Level Medical Clinic: Protocol for a Validation Study

**DOI:** 10.2196/10900

**Published:** 2018-12-10

**Authors:** Mapa Mudiyanselage Prabhath Nishantha Piyasena, Venkata S Murthy Gudlavalleti, Clare Gilbert, Jennifer LY Yip, Tunde Peto, David MacLeod, Charith Fonseka, Aruna Kulatunga, BGWMKCB Bandutilake, Mangala Dhanapala, Lalani Pathirana, Heshani Dissanayake

**Affiliations:** 1 Clinical Research Department International Centre for Eye Health London School of Hygiene and Tropical Medicine London United Kingdom; 2 School of Medicine, Dentistry and Bio-medical Science Queen's University Belfast Ireland; 3 Vitreo-retina Unit National Eye Hospital Colombo Sri Lanka; 4 National Hospital of Sri Lanka Colombo Sri Lanka

**Keywords:** diabetes, diabetic retinopathy, digital imaging, hand-held camera, mydriatic, nonmydriatic, physician grader, screening, Sri Lanka

## Abstract

**Background:**

Visual impairment and blindness from diabetic retinopathy (DR), which can be reduced by early screening and treatment, is an emerging public health concern in low-income and middle-income countries (LMICs) owing to the increasing prevalence of diabetes mellitus (DM). However, no systematic screening exists in most LMIC settings. The Western province of Sri Lanka has the highest prevalence of DM (18.6%) in the country. A situational analysis identified a marked gap in DR screening (DRS) and treatment services uptake in this region; only opportunistic screening is practiced currently.

**Objective:**

The aim of this protocol is to describe the methods of development and validation of a DRS intervention using a hand-held nonmydriatic digital camera by physician graders in a non-ophthalmological setting at a tertiary-level medical clinic to propose a valid and feasible modality to improve uptake.

**Methods:**

DRS modality was developed after assessing barriers and identifying the most appropriate personnel, methods, and location for screening services, following formative research work. The validation will be conducted in a public sector tertiary care center in the Western province of Sri Lanka. The selected physicians will be trained on capturing and grading images according to a valid locally adopted protocol. Two physicians rated high on training will screen a sample of 506 people with DM at a medical clinic. They will use nonmydriatic and mydriatic 2-field imaging strategy. The validity of the proposed screening procedure will be assessed and compared with the mydriatic indirect biomicroscopic examination by a senior retinologist.

**Results:**

The validity of screening by physician graders will be analyzed and the sensitivity, specificity, and predictive values (with 95% CIs) calculated by the dilation status and for each grader. The diagnostic accuracy at each level of severity of DR will be assessed to define the most appropriate referable criteria. Data is currently being collected.

**Conclusions:**

The outcome of this study will be useful for the detection of a defined level of DR at non-ophthalmological setting to filter the people with DM before referral to an eye clinic. This will be helpful to improve the uptake and identify risk groups in advance to prevent sight-threatening DR. Furthermore, evidence from this study will be useful for the implementation of a DRS program in this region and in similar communities.

**International Registered Report Identifier (IRRID):**

PRR1-10.2196/10900

## Introduction

The prevalence of diabetes mellitus (DM) and the number of people affected by DM is increasing rapidly in all regions. The International Diabetes Federation estimated that 425 million people had diabetes in 2017, which will increase to 629 million in 2045 globally [1]. This increase is expected to be the highest in low-income and middle-income countries (LMICs) compared with high-income countries (HIC) [2]. Diabetic retinopathy (DR) is a common microvascular complication of DM, which can lead to visual impairment and blindness if not detected early and treated [3]. Many studies have reported that visual loss from DR can be largely prevented by early screening and appropriate treatment [4-6]. Diabetic retinopathy screening (DRS) can be done in 2 ways, systematic screening similar to national-level programs in HIC versus opportunistic screening and case detection, which is common in low-income settings. Most LMICs are unlikely to have full population-based screening program owing to resources constraints. The current method of DRS in most LMICs is direct ophthalmoscopy, which has a lower diagnostic accuracy and found to be ineffective even after training [7]. The mydriatic biomicroscopic examination by an ophthalmologist is practically not possible in these countries owing to a low number of ophthalmologists, and eye clinics are overburdened with highly prevalent blinding conditions such as cataract [8].

The reasons for the unavailability of DRS programs (DRSPs) in LMIC settings are mostly attributed to the lack of skilled human resources, financial resources, and evidence of what works in the local system [9-11]. Therefore, it would be important to understand the approaches for screening, especially in non-ophthalmological settings. Conventional digital cameras need a larger space, skilled photographers, and large image storage devices. In addition, systematic screening using sophisticated table-top imaging systems incur high capital investment though they are cost-effective [12]. Hand-held digital cameras are easy to move, require minimum space, minimum power consumption, and are user-friendly [13]. In addition, nonmydriatic hand-held cameras are less discomforting to participants and can be used while people with DM are waiting in front of a physician for consultation. The usage of a camera without pupil dilatation is comfortable to people with DM, as well as easy for providers. However, the latter depends on the quality of the image available for grading [14].

Various photographic studies have looked at the diagnostic test accuracy of DRS using digital imaging. Most of these studies used static table-top imaging systems and were conducted in HICs. These studies have shown a sensitivity of 68%-97% and a specificity of 71%-100% in nonmydriatic imaging using ophthalmic human resources as index graders [15-18]. Similarly, in mydriatic imaging, most of the studies have used table-top imaging systems, ophthalmic human resources as index test graders, and were conducted in HICs. These studies have shown a sensitivity of 77%-97% and a specificity of 76%-98% in mydriatic digital imaging [19-22]. There is a gap in evidence in digital retinal imaging in LMICs using non-ophthalmic human resources. In addition, the usage of context-specific imaging systems, such as hand-held digital retinal camera, in non-ophthalmic setting was not reported in the current literature.

Sri Lanka has achieved remarkable development in the health sector; however, there are public health concerns such as DR which have not been addressed to date [23]. The crude prevalence of DM in Sri Lanka was 12.6% (>20 years), being highest in the Western province (18.6%, 95% CI 15.8%-21.5%) [24]. In the Western province, there are approximately 750,000 (age>18 years) people with DM, 20% (150,000/750,000) of whom are likely to have nonproliferative DR (NPDR). A situational analysis conducted in this region has shown that the number of people undergoing opportunistic screening and free treatment in the public sector was far lower than the estimated need [25]. There is no systematic DRS in the Western province despite the high prevalence of DM [25]. In addition, there is no published data on this topic from Sri Lanka. The aim of this protocol is to describe the methods of validation of a DRS approach using digital imaging by physician graders in a tertiary-level public sector medical clinic. This study will demonstrate the functional and technical feasibility of using a hand-held digital camera in an LMIC non-ophthalmological setting and assess the diagnostic accuracy.

## Methods

### Ethics Approval

Ethics review committees of National Eye Hospital (Colombo, Sri Lanka) and London School of Hygiene & Tropical Medicine (United Kingdom) granted ethics approval.

### Development of the Diabetic Retinopathy Screening Modality and Training

**Figure 1:**
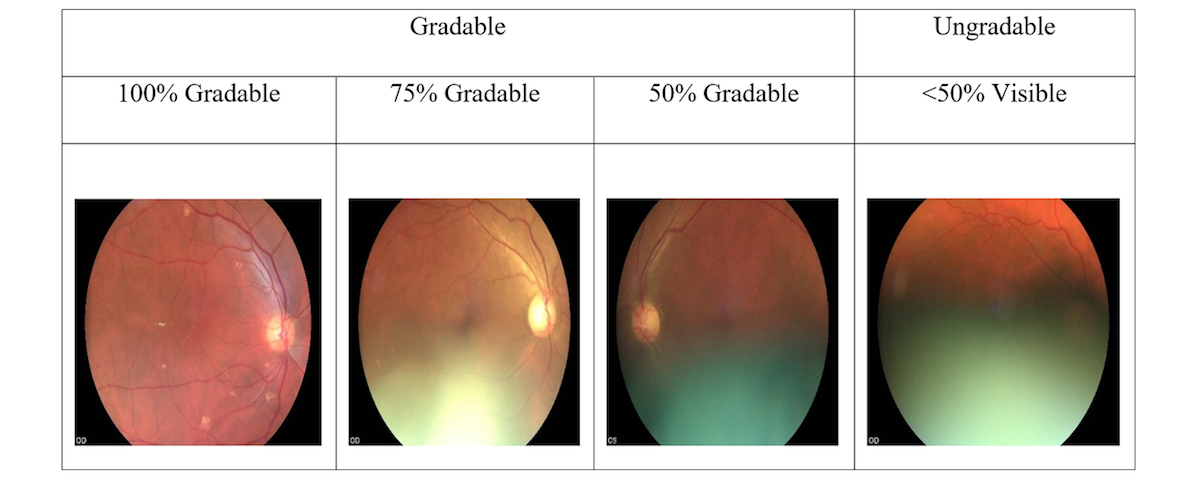
Evaluation of image quality-levels of gradeability based on the proportion of the image that can be graded.

The initial formative research showed that nonmydriatic digital retinal imaging at medical clinics by general physicians was a potential option for the local setting. We selected 9 general physicians from a tertiary-level institution following informed consent, and they underwent a competency-based training by 2 retinologists from a tertiary center, which included the following: capturing retinal fields using a hand-held fundus camera, identification of signs of DR (including macular signs) using images, and DR grading according to an adapted classification system (Table 1). DR signs are graded at 4 levels as follows: none=R0, mild NPDR=R1, moderate NPDR=R2, severe NPDR=R3, and proliferative DR and above=R4. Macular changes are graded as follows: none=M0 (maculopathy absent) and exudate(s) or blot hemorrhage(s) within 2-disc diameters from the center of the fovea=M1 (maculopathy present). Guidelines were used to standardize reporting of image quality, which included ungradable images based on the proportion of the retina visible for grading (Figure 1). After the training, physicians were tested using a set of standard images of DR, and the two physicians who reached the required level of agreement with the retinologists (κ=.8-.9) were selected as graders in the validation study.

**Table 1:** Adapted diabetic retinopathy classification for the validation study.

Signs	No DR^a^ (R0)	Mild BDR^b^ or NPDR^c^ (R1)	Moderate BDR or NPDR (R2)	Severe NPDR (R3)	PDR^d^ (R4)
Microaneurysms	No	Few	Multiple	Multiple	Present
Hard exudates^e^	No	Few	Multiple	Multiple	Present
Cotton wool spots	No	Occasional	Multiple	Multiple	Present
Intraretinal hemorrhage^e^	No	Few	>20 in 1-3 quadrants	>20 in 4 quadrants	Present
Venous beading	No	Occasional	Present in 1-2 quadrants	Present in >2 quadrants	Present
IRMA^f^	No	No	Present ~1 quadrant	Prominent >1 quadrant	Present
NVD^g^	No	No	No	No	Present
NVE^h^	No	No	No	No	Present
Vitreous or preretinal hemorrhage	No	No	No	No	Present—advanced PDR
Traction	No	No	No	No	Present—advanced PDR
Fibrosis	No	No	No	No	Present—advanced PDR

^a^DR: diabetic retinopathy.

^b^BDR: Background DR.

^c^NPDR: nonproliferative DR.

^d^PDR: proliferative DR.

^e^Not within the definition of maculopathy.

^f^ IRMA: Intraretinal microvascular abnormalities.

^g^NVD: Neovascularizations over the disc.

^h^NVE: Neovascularizations elsewhere.

### Sample Calculation and Recruitment

The sample size (n=405) was calculated on the basis of 95% CIs, 10% margin of error, expected sensitivity 70%, and the prevalence of moderate NPDR among people with DM of 20%. Then, we inflated the sample size by an additional 25% (n=101, total n=506) to take account of ungradable images. An interim analysis will be undertaken to ascertain the level of ungradable images (ie, <50% of the retina visible) and the sample size increased, if required.

**Figure 2:**
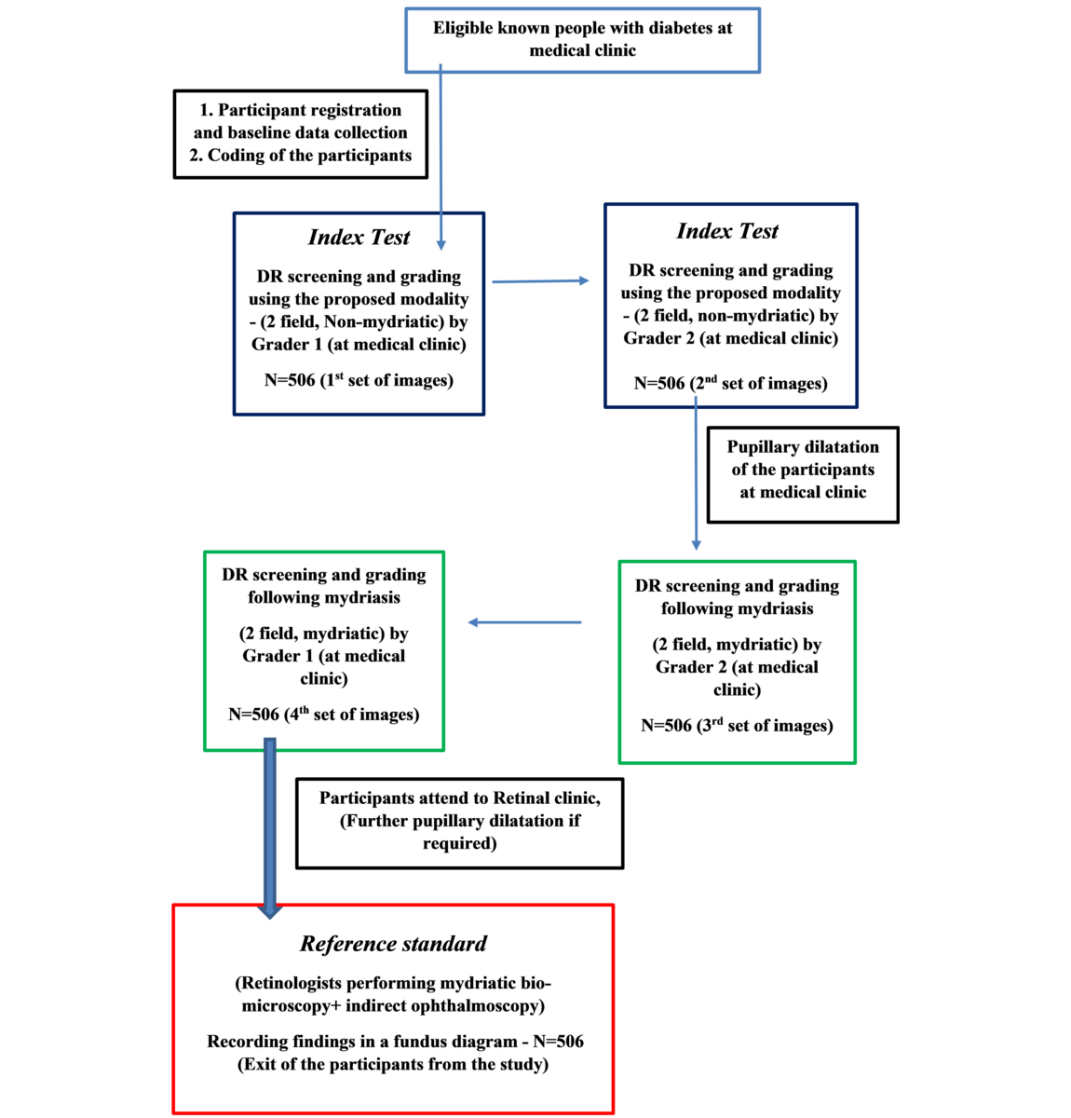
Participants' flow diagram in the validation.

This study is a prospective observational study by design. A consecutive sample of diagnosed people with DM (age >18 years) without previous DRS at an eye clinic will be eligible to participate, after giving written informed consent. Eligible participants will be recruited by trained research assistants when people with DM present for routine medical care at the main tertiary center in Colombo. People with DM with previous retinal screening, DR-related treatment (laser treatment, intravitreal injections, and pars plana vitrectomy), and those who were currently under any DRSP or treatment will be excluded from the study. Figure 2 shows the participants’ flow diagram. Participants’ characteristics will be documented in a questionnaire schedule by research assistants on recruitment.

**Figure 3:**
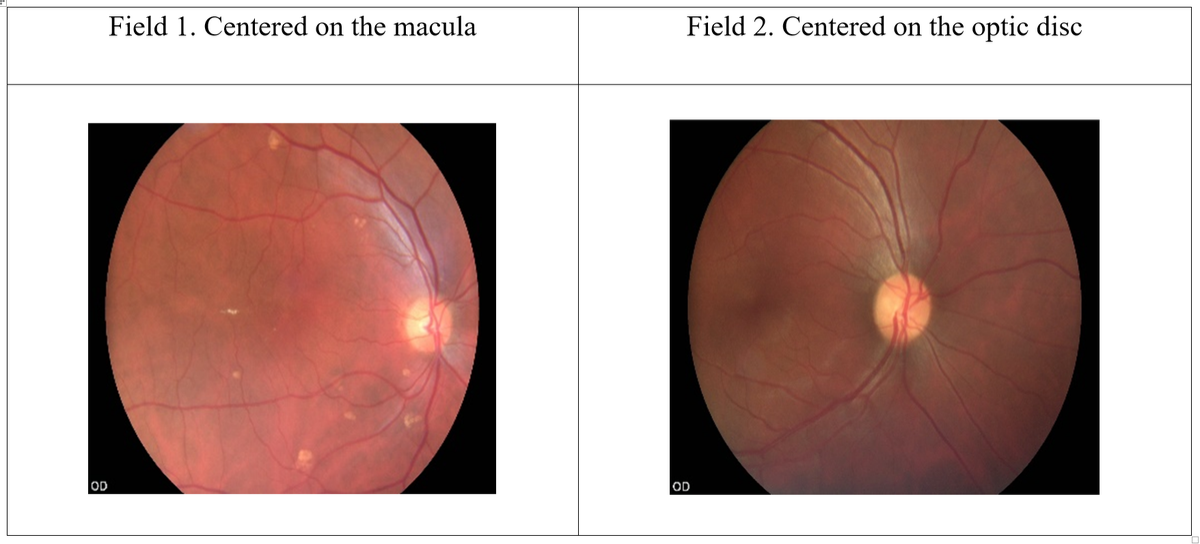
Two retinal fields captured.

### Imaging System, Capturing Images, and Grading

Two-field nonmydriatic and mydriatic retinal images will be captured and stored (Figure 3). Participants will undergo digital retinal imaging (using VISUSCOUT100 hand-held nonmydriatic fundus camera-2017; Carl Zeiss, Germany) by the physician graders at the time of presentation. This imaging system has a 40° field-of-view with 5 megapixels (type of camera sensor: complementary metal-oxide semiconductor; resolution 800×480) and captures color and red-free images in a focus range of −20 D (diopters) to +20D. The minimum pupil size required is 3.5 mm, and 9 light-emitting diodes are available for internal fixation.

First, 2-field (first field-macula centered, second field-disc centered; Figure 3), 40° retinal images will be captured in each eye by each physician grader without pupillary dilatation. Subsequently, participants’ pupils will be dilated using 2.5% phenylephrine, and the same fields will be captured, following adequate mydriasis (5-6 mm).

Each set of images will be coded and stored by research assistants after capturing. The coded image sets will be given back to the same physician graders for grading. During grading, nonmydriatic images will be graded first. The graders will be masked to the history and clinical examination findings. The retinopathy and macular signs will be identified and entered by physician graders into a hardcopy data table. Finally, it will be entered into a Microsoft Excel datasheet by research assistants. The grading data consistency checks and cleaning will be done by an independent statistician.

### Reference Test

The reference test will entail a detailed, dilated fundus examination by an experienced retinologist using slit-lamp biomicroscopy with a 90D lens and indirect ophthalmoscopy using a 20D lens. After imaging, this examination will take place as early as possible. The retinologist will be masked to the clinical status and physician graders’ findings. In addition, a detailed anterior segment examination (clarity of cornea and status of the lens) and media (vitreous) examination will be done by the reference test grader. The lens opacity will be graded according to the lens opacity classification system, version 111.

### Quality Assurance and Agreement Analysis

For quality assurance, 15% of each nonmydriatic and mydriatic image sets will be evaluated by the retinologist for technique, ability to image the required field, and gradeability. Then, 15% of each hundred image sets will be given back to physician graders for double grading to assess the repeatability and intragrader agreement in the first and second attempts of grading images. A sample of the same image sets (n=200) will be graded by the retinologist to calculate the intergrader agreement.

### Data Analysis

We will analyze the validity of screening by physician graders and calculate the sensitivity, specificity, and predictive values with 95% CIs for each method of screening and by the grader. The analysis will be conducted by including and excluding ungradable images and considering each eye as a unit of analysis and by a person considering the worst eye. Intragrader and intergrader agreement (kappa) for both mydriatic and nonmydriatic index tests will be calculated and compared with the findings by the retinologist. A subgroup analysis will be conducted for the identification of the presence or absence of DR (any DR), moderate NPDR, and above with or without macular signs to make recommendations for a referable criterion for the local context in DRS by physician graders.

## Results

The physician graders have been trained, and currently, validation is being done in the Western province of Sri Lanka. The results of this study will be published in detail according to the Quality Assessment of Diagnostic Accuracy Study guidelines [26]. Data will be entered using a Microsoft Excel (2016) worksheet and transferred into STATA/IC-v14.2 analytical package following cleaning, consistency checks, and analysis. The sensitivity, specificity, and predictive values for each strategy and each level of DR will be presented using the same variables of 2 physician graders (nonmydriatic and mydriatic separately) compared with the reference standard, along with 95% CIs.

## Discussion

The level of skills acquired by physician graders is an important factor in the screening outcome. Different non-ophthalmologist graders have successfully conducted DRS in some settings [27-29]. We will describe the diagnostic accuracy of the detection of DR by physician graders. In addition, we will be able to study the effect of a range of population characteristics on the validity of detecting DR using imaging and understand the role of non-ophthalmic personnel to make recommendations for a systematic DRSP. In addition, we will describe the referral criterion applicable to this local context based on the validation study results. Defining a referable level DR at a non-ophthalmological setting, in a context where there is no systematic DRS, will filter out those not needing a referral and therefore reduce the workload at an ophthalmologist’s clinic. The 7-field imaging strategy used in early treatment diabetic retinopathy study is considered as the gold standard in DRS [30]. However, this technique is practically not feasible in this context owing to resources constraints. Therefore, we proposed to use the locally accepted reference standard of retinologists’ examination as the suitable reference standard. Digital retinal imaging has previously shown diagnostic accuracy levels that would comply with the accepted standards of established national-level screening programs [15,22,31].

A few studies (conducted in HICs) have used non-ophthalmologist human resources in DRS, with which we could compare our results. In Singapore, a nonmydriatic fundus camera showed a sensitivity of 69.8% (95% CI 61.3%-77.2%) and a specificity of 94.4% (95% CI 92.3%-96.1%) for nonphysician graders using a single field [32]. A study in the United Kingdom on DRS by general practitioners using 35-mm color images showed that detecting any level of DR increased from 62.6% (95% CI 55.9%-69.4%) with direct ophthalmoscopy to 79.2% (95% CI 73.6%-84.9%) using retinal photographs, and specificity remained unchanged (direct ophthalmoscopy 75.0% [95% CI 69.5%-80.5%] vs 73.5% [95% CI 68.0%-79.1%]) [33]. They concluded that retinal photography by trained general practitioners in primary care settings could attain an acceptable level of detection of sight-threatening DR (87%) [33]. In Thailand, the use of single-field digital nonmydriatic imaging showed a sensitivity of 80% and a specificity of 96% in a sample of people with DM, where 54.7% people with DM were aged 41-60 years and 45.3% people with DM had diabetes since 1-5 years [34].

Another important consideration in this study would be the gradeability of images. The image gradeability will depend on the lens opacity, media opacity, pupil size, and reflectivity of the fundus. We envisaged poor gradeability in nonmydriatic imaging considering the high prevalence of cataract in this local setting. Furthermore, iris color, age, and other population characteristics may affect the quality of images [14]. Scanlon et al showed that in the >80-years age group, the technical failure rates reduced from 41.6% to 16.9% following mydriasis. This study concluded that the odds of having one eye ungradable increases by 2.6% (95% CI 1.6%-3.7%) for each extra year since the diagnosis of DM and major cause of ungradability was having central cataract (57%) [35]. We will describe the factors affecting gradeability of images in addition to the diagnostic test accuracy results.

In this study, we will demonstrate the diagnostic accuracy of physician graders compared with the retinologist to make recommendations for developing an integrated DRSP in LMICs where there is no systematic DRS. The outcome of this study will be useful for the implementation of a systematic DRSP in this region and similar communities.

## Abbreviations

DM: diabetes mellitus
DR: diabetic retinopathy
DRS: diabetic retinopathy screening
DRSP: diabetic retinopathy screening program
HIC: high-income country
LMICs: low-income and middle-income countries
NPDR: nonproliferative diabetic retinopathy
